# Double Aortic Arch in an Asymptomatic Adult

**DOI:** 10.7759/cureus.37437

**Published:** 2023-04-11

**Authors:** William J Lee, Yash K Shah, Albert Ku, Nidhi R Patel, Pierre D Maldjian

**Affiliations:** 1 Radiology, Rutgers University, Newark, USA; 2 Radiology, Drexel University College of Medicine, Philadelphia, USA

**Keywords:** esophageal compression, tracheal compression, adult male, incidental radiological finding, vascular ring, double aortic arch

## Abstract

We present a rare double aortic arch (DAA) diagnosis incidentally on CT in a 60-year-old male who presented with pneumonia. DAA is a vascular ring that typically manifests in infants or children due to compression of the esophagus or trachea, resulting in dysphagia or dyspnea. Diagnosis of DAA in adulthood is usually due to the delayed emergence of obstructive symptoms. We present a case of DAA in an adult patient without dysphagia or dyspnea. We discuss factors that can lead to the presentation of DAA in adults. These include an absence of associated congenital disabilities, insufficient tracheal or esophageal constriction in childhood and the onset of compressive symptoms later in life from decreased vascular compliance.

## Introduction

Double aortic arch (DAA) is a rare congenital anomaly caused by failure of the right component of the fourth aortic arch to regress during embryogenesis leading to the persistence of a right-sided aortic arch in addition to the normal left aortic arch. The double arches form a complete vascular ring when they unite as the descending thoracic aorta. The incidence of DAA is about one in 15,000 births [1]. DAA presents earlier in life than other forms of vascular rings due to the entrapment of the trachea and esophagus. Symptoms such as dysphagia, vomiting, respiratory distress, wheezing, and stridor can indicate the presence of a DAA in a newborn or child [2]. DAA can be diagnosed in adults if obstructive symptoms arise [3]. However, we present a case of DAA in an adult as an incidental finding without obstructive symptomatology and discuss factors that can lead to delayed presentation of this disorder.

## Case presentation

A 60-year-old man presented to the emergency department complaining of a worsening cough and fever over the prior three weeks. The patient reported multiple sick contacts with similar symptoms. He denied shortness of breath, choking sensations, difficulty swallowing, calf pain, peripheral edema, or recent travel.

**Figure 1:**
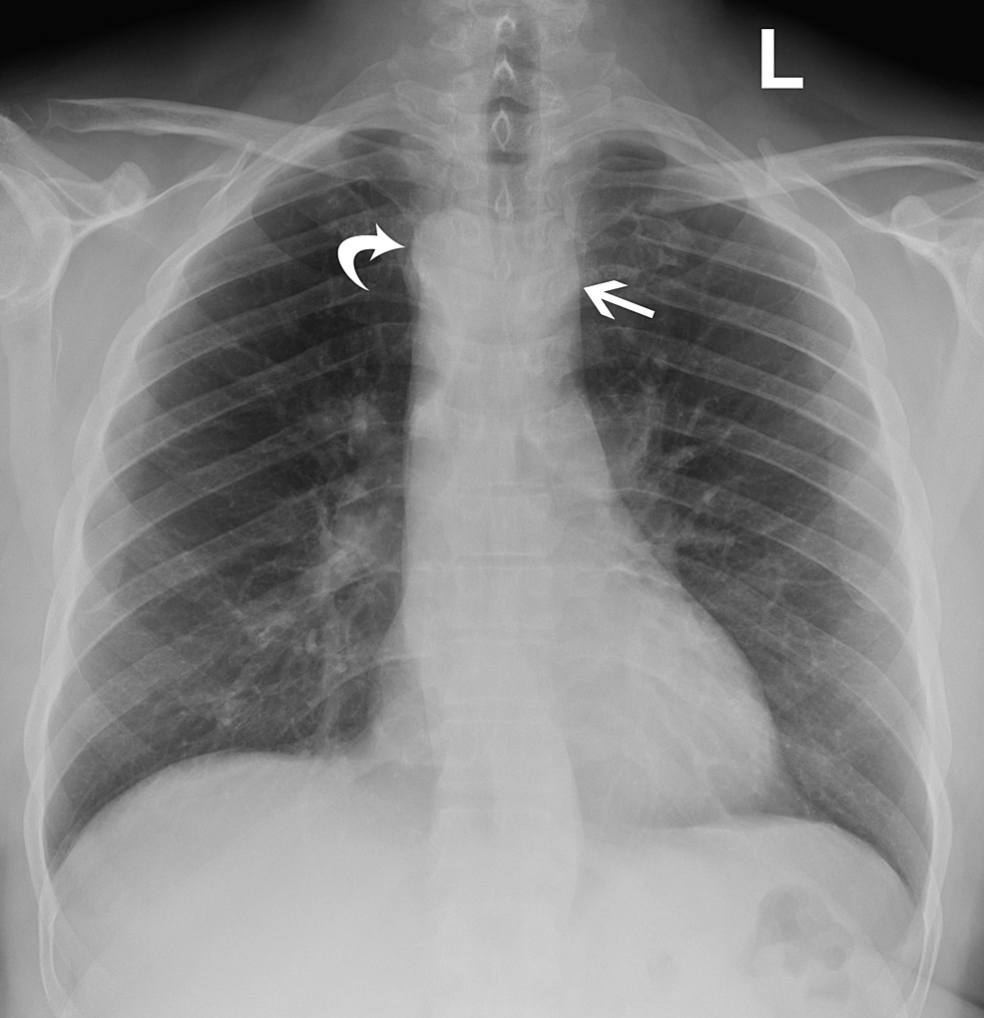
Posteroanterior chest radiograph shows right (curved arrow) and left (straight arrow) paratracheal opacities representing a double aortic arch.

The patient was afebrile on initial evaluation with a heart rate of 82, respiratory rate of 22, blood pressure of 155/78 mmHg, and an oxygen saturation of 95% on room air. On physical examination, no wheezing, crackles, or rhonchi were present on the auscultation of the lungs, and there was no peripheral edema or calf tenderness. Relevant laboratory tests showed a normal white blood cell count of 8.2 (reference range 4.0-11.0 K/uL) and a negative respiratory panel. EKG showed normal sinus rhythm. The chest radiograph did not reveal any consolidations, although it did show paratracheal opacities subsequently demonstrated to represent a double aortic arch (Figure 1).

**Figure 2:**
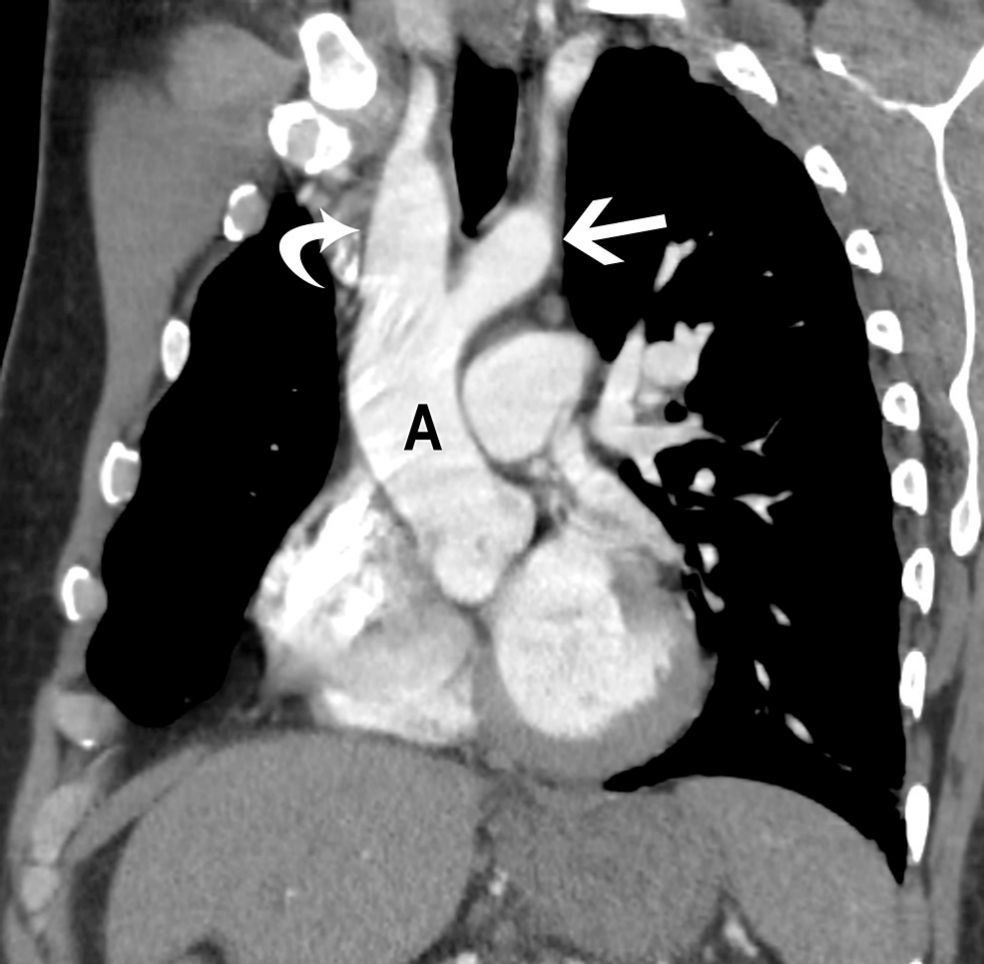
Coronal reformatted image from contrast-enhanced computed tomography scan of the chest shows the ascending aorta (A) splitting into the right (curved arrow) and left (straight arrow) aortic arches.

**Figure 3:**
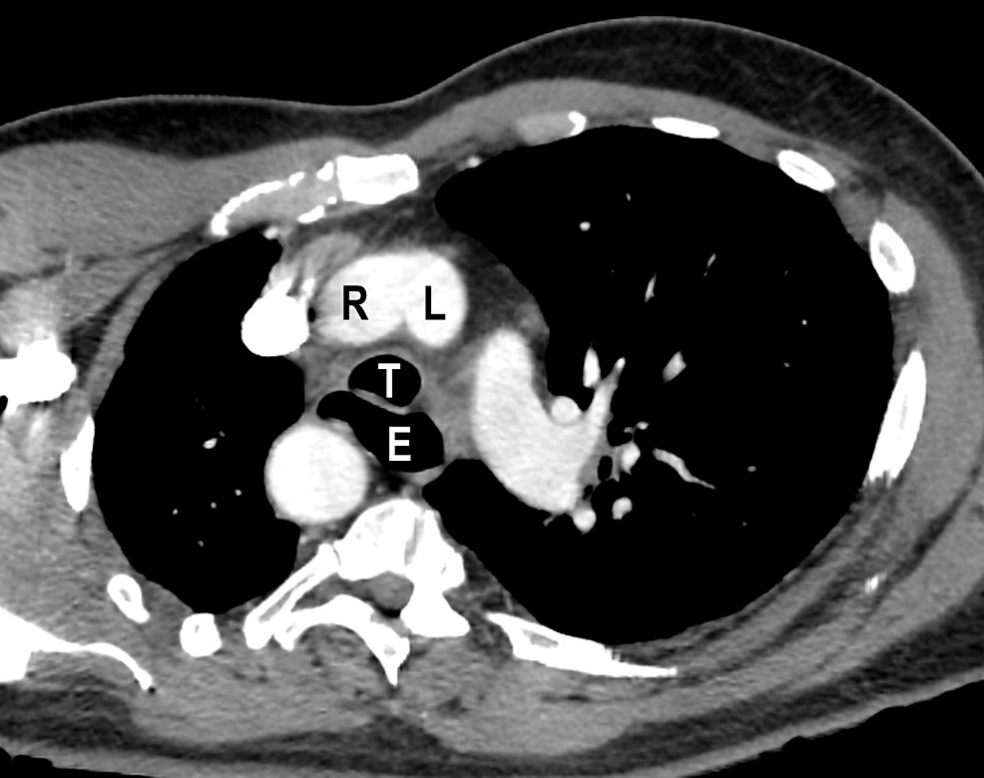
Oblique axial reformatted computed tomography image shows the ascending aorta splitting into the right (R) and left (L) limbs of the double arch anterior to the trachea (T). Note that the esophagus (E) is mildly distended at this level.

**Figure 4:**
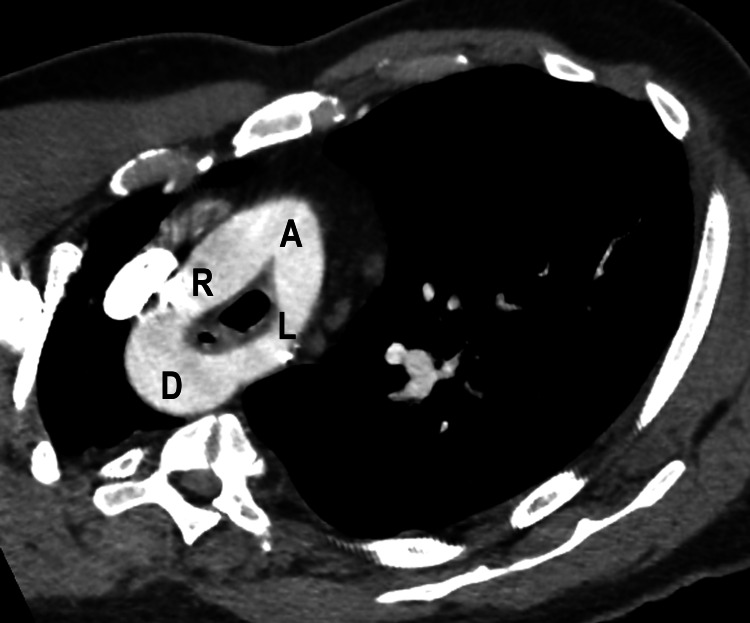
Oblique axial reformatted computed tomography image depicts a vascular ring with the ascending aorta (A) dividing into the right (R) and left (L) arch components which merge to form the descending thoracic aorta (D). Note how the ring surrounds the trachea and constricts the esophagus.

On re-evaluation of the patient, an oxygen saturation of 93% on room air was noted. Due to concern for pulmonary embolism, CT angiography of the chest was obtained. The contrast-enhanced CT scan of the chest showed no evidence of pulmonary embolism, although there were foci of airspace consolidation in the bilateral lower lobes concerning pneumonia. As an incidental finding, the CT demonstrated a double aortic arch producing mild compression of the esophagus (Figures 2-4 and Videos 1-2). The right arch was higher and slightly larger than the left arch, with the descending thoracic aorta on the right.

**Video 1:** Volume-rendered images from a CT scan depicts a double aortic arch.

**Video 2:** Volume-rendered images from a CT scan with the segmentation of the airways (red) and esophagus (green) depict the relationship of the double aortic arch to those structures. Note compression of the esophagus by the left limb of the double arch.

The patient was given a presumptive diagnosis of pneumonia and was discharged on oral antibiotics. The patient recovered, and the follow-up was uneventful.

## Discussion

DAA is exceedingly rare, with one in 15,000 births accounting for only 1% of all congenital heart diseases [4]. The majority of DAA cases are diagnosed in infancy or childhood. The DAA's compression of the esophagus or trachea results in the typical symptoms of dysphagia, vomiting, dyspnea, wheezing, or stridor [5]. Presentation of DAA in an adult can occur from symptoms similar to those in childhood, especially from dysphagia due to compression of the esophagus. Reports of asymptomatic adults with DAA (such as our case) are sparse in the literature [4-6]. Certain factors increase the likelihood of diagnosing DAA in adulthood rather than childhood. These include the absence of other congenital anomalies, insufficient compression of the trachea or esophagus by the vascular ring in childhood, and the onset of compressive symptoms in adulthood due to loss of vascular compliance.

DAA has been associated with cardiac and other congenital anomalies [6]. DiGeorge syndrome, a chromosome 22q11 deletion, is observed in approximately 20% of DAA cases. Congenital heart defects such as tetralogy of Fallot, ventricular septal defect, and truncus arteriosus can also be associated with DAA [7]. Congenital anomalies in children would result in the workup and prompt diagnosis of DAA if also present. DAA is usually an isolated anomaly if first diagnosed in an adult.

Symptoms like respiratory distress and dysphagia occur in infancy when the vascular ring significantly compresses the trachea or esophagus. Mild compression of the esophagus may not be sufficient to result in obstructive manifestations [8]. In our case, there was evidence of mild compression of the esophagus by the vascular ring, although not enough to be detrimental to the patient. As adults with DAA age, constriction of the esophagus may progress from loss of vascular compliance resulting in delayed onset of symptoms. Causes of arterial stiffening include atherosclerotic plaque deposition, systemic hypertension, decreased connective tissue elasticity, and decreased smooth muscle relaxation [9-11].

CT angiography is the modality of choice for diagnosing DAA due to the availability of multiplanar image reformatting and volume rendering techniques [12]. With three-dimensional volume rendering, the aortic arches can segment the esophagus and trachea to depict their spatial relationships and extent of compression. Our case demonstrates the typical appearance of a double aortic arch, with the right arch being higher and larger than the left arch and the subclavian and carotid arteries arising symmetrically from their ipsilateral arch components.

Treatment of DAA depends on the severity of the symptoms. Patients with only mild esophageal or tracheal compression with no signs of respiratory distress or dysphagia are managed conservatively, as in our case. More severe symptoms may require surgical intervention with a vascular ring division to relieve the compressive effects. The smaller aortic arch is usually divided and ligated, leaving the larger, more dominant arch functionally intact [13].

## Conclusions

We present a case of DAA in an older male patient without the typical symptoms of dysphagia or dyspnea. Although diagnosing DAA in adults is infrequent, certain factors can delay diagnosis. These include the absence of other congenital anomalies, insufficient compression of the trachea or esophagus by the vascular ring in childhood, and the onset of compressive symptoms in adulthood due to loss of vascular compliance. We also illustrate the utility of CT with volume rendering and segmentation of structures for depicting the anatomical relationships of this vascular anomaly to the trachea and esophagus.
